# Association Between MTHFR Genetic Variants and Multiple Sclerosis in a Southern Iranian Population

**Published:** 2015

**Authors:** Fakhraddin Naghibalhossaini, Hesam Ehyakonandeh, Alireza Nikseresht, Eskandar Kamali

**Affiliations:** 1 *Department of Biochemistry, Shiraz University of Medical Sciences, School of Medicine, Shiraz, Iran.*; 2 *Autoimmune Research Center, Shiraz University of Medical Sciences, School of Medicine, Shiraz, Iran.*

**Keywords:** Multiple sclerosis, MTHFR, SNP, genotype

## Abstract

Multiple sclerosis (MS) is a demyelinating neuro- inflammatory autoimmune disease of the central nervous system. Genetic predisposition has long been suspected in the etiology of this disease. The association between *MTHFR* polymorphisms and MS has been ivestigated in different ethnic groups. We investigated the association between *MTHFR C677T* and *A1298C* missense variants and MS in 180 patients and 231 age- and gender-matched healthy controls in a Southern Iranian population. The mutagenically separated PCR (MS-PCR) and PCR-RFLP methods were used to genotype *MTHFR* at position 677 and 1298, respectively. Compared with controls, we observed a strong association between two MTHFR variants and the risk of developing MS. Subjects carrying 677*T* allele (*CT* and *TT* genotypes) had increased susceptibility to MS as compared to those carrying *CC *genotype (odds ratio (OR) for *CT*= 2.9, 95% confidence interval (95% CI)= 1.88-4.49; OR for *TT*= 6.23, 95% CI= 3.08-12.59). The variant *1298AC* genotype also increased the risk for MS among our study population (OR= 2.14, 95% CI= 1.37-3.34). Combined genotype analysis for two *MTHFR* SNPs revealed that compared to the wild type genotypes (*677CC/1298AA*), 3 genotypes including *TT/AC*, *CT/AC*, and *TT/AA* were significantly at increased risk for MS development (OR= 13.9, 5.3, and 4.9, respectively). Our results suggest a possible gene dose- dependent association between MTHFR mutrant alleles and the risk of MS development.

Multiple sclerosis (MS) is a complex autoimmune disease of the central nervous system (CNS) resulting in CNS inflammation and demyelination of nerve axons (1, 2). A significant increase in MS incidence has been reported in Iran over the last decade, especially in females (3).

The MS etiology is not well-understood; however, several studies suggest that environmental and genetic factors might be involved in the etiology of this disease (4, 5). Age-dependant exposure to viral infection may also play a role in MS susceptibility (6).

Previous studies have indicated that MS patients have elevated plasma and cerebrospinal fluid (CSF) levels of neurotoxic amino acid homocysteine (7). Other studies have suggested that hyperhomocystenemia is a risk factor for dementia and Alzheimer’s disease (8, 9) and might be associated with cognitive impairment in MS patients (10). Increased blood homocysteine was shown to be associated with the sensitization of neurons to oxidative stress that promotes apoptosis and hypersensitivity to excitotoxicity (11). Homo-cysteine may induce neurotoxicity through the oxidation of sulfhydryl groups resulting in generation of reactive oxygen species (11) and overstimulation of N-methyl-D-aspartate (NMDA) receptors resulting in neuronal damage due to excessive Ca^2+^ influx (12).

Methylenetetrahydrofolate reductase (MTHF-R) is a key folate metabolizing enzyme that functions at the junction between two critical pathways regulating one carbon metabolism, nucleotide synthesis and synthesizing the universal methyl donor S-adenosyl methionine (SAM). MTHFR gene is polymorphic and two common non-synonymous mutations, *C677T* (A222V; rs1801133) and *A1298C* (E429A; rs1801131), have been associated with decreased enzyme activity and the increased levels of plasma homocysteine (13-16). As a result, the *MTHFR* genotypes may play a role in MS susceptibility. Both of the above-mentioned *MTHFR* polymorphisms have been extensively studied for associations with several diseases including neural tube defects (15, 17), and cardiovascular disease (18, 19). A few studies have also investigated the relationship between these polymorphisms and MS (20-22). The aim of this study was to investigate the association between functional polymorphisms of the *MTHFR *gene with MS among Southern Iranian population.

## Materials and methods

**Study population**

This case- control study consisted of 180 unrelated patients and 231 healthy controls. The MS population was obtained from patients in university hospitals in Shiraz, Southern Iran, and the diagnosis was made by a neurologist according to the revised McDonald criteria (23). The associated MS population was comprised of three clinical subtypes: 128 relapsing–remitting MS (RR-MS; 71.1%), 43 secondary progressive MS (SP-MS; 23.9%), and 9 primary progressive MS (PP-MS; 5%). The control group was also obtained from healthy volunteers from the general population, which had been matched for age, gender, and ethnicity. Ethics approval for experimentation on humans was obtained from the Institutional Ethics Committee.

**Genotype analysis**

Genomic DNA was extracted from peripheral blood using a standard salting-out procedure (24). Genotyping of *MTHFR* at position 677 of DNA from healthy subjects (control) and multiple sclerotic patients was performed using the mutagenically separated PCR (MS-PCR) method, as previously described (25). The *A1298C* mutation of *MTHFR *was also examined by PCR-RFLP of DNA samples using the enzyme MboII (MBI Fermentas, Lithuania) as described previously (26).

**Statistical analysis**

All statistical analyzes were performed using the SPSS version 16 software package (SPSS Inc., Chicago, IL). Genotype and allele frequencies for the *MTHFR *genotype variants were investigated using standard Chisquare (χ^2^) analysis. In addition, conditional multivariate logistic regression analysis for matched case- control groups was used to calculate odds ratio (OR) and 95% confidence intervals (95% CI). A p-value <0.050 was considered as statistically significant.

## Results

We investigated the association between two common functional polymorphisms of *MTHFR *(*C677T* and *A1298C*) and MS incidence among Iranian patients. The case-control populations consisted of 180 patients and 231 healthy controls. The cases were more likely to be females (74%) older than 26 years (54.4%). The mean age of patients was 26.0± 12.3 years. The groups of patients and controls did not significantly differ concerning gender or age.

**Fig. 1:**
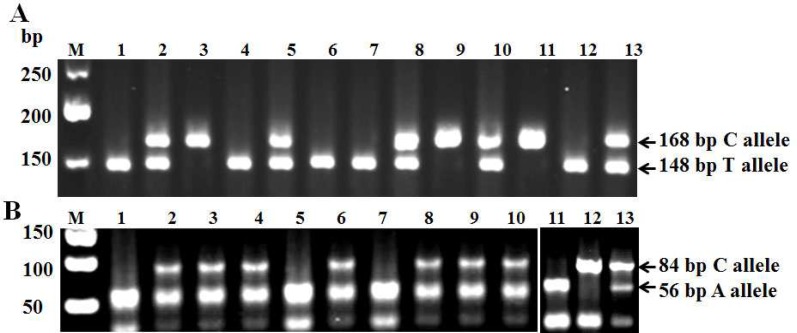
Representative examples of genotyping of *MTHFR* positions 677 and 1298. **A. **MS-PCR assay for the genotyping of *MTHFR C677T* polymorphism. The *677C *alleles (168 base pair product) were separated from the *677T *alleles (148-base pair product) by electrophoresis on a 2.5 % agarose gel. **B.** PCR-RFLP assay for genotyping of *MTHFR A1298C* polymorphisms. Digestion of the 163 bp PCR product of the 1298A allele yields five fragments of 56, 31, 30, 28, and 18 base pairs, whereas the 1298C allele results in four PCR bands of 84, 31, 30, and 18 base pairs. The digested PCR products were separated by electrophoresis on a 2.5% agarose gel. The three possible genotypes are discernible by detection of the 84and 56 bp fragments

*MTHFR* *C677T* and *A1298C *genotyping was performed by MS-PCR and PCR-RFLP methods, respectively. Illustrative examples of genotype analysis of the two *MTHFR *variant genotypes are shown in Fig. 1. The results of genotype frequencies and odds ratios for *MTHFR* genotypes and MS are presented in Table 1. There was no significant HardyWeinberg disequilibrium concer-ning the *MTHFR* 677 and 1298 genotypes in controls. The distribution of *MTHFR* 677 genotypes among patients also agreed with that expected from the Hardy-Weinberg equilibrium (χ^2^= 0.5, P= 0.47). However, significant departures from Hardy-Weinberg equilibrium were observed for *MTHFR *1298 genotypes among cases (P= 0.00).

In our study, the allele frequency distributions of *MTHFRC 677T* were significantly different between patient and control groups (41.9% versus 20.3%, P= 0.00). The frequencies of the MTHFR *C677T* genotypes in the patients (*CC*, 35%; *CT*, 46.1%; *TT*, 18.9%) were also significantly different from controls (*CC*, 65%; *CT*, 29.5%; *TT*, 5.5%) (P= 0.00; Table 1). The MS patients were presented with higher homozygous *TT* genotype than control group, as manifested by an odds ratio of 6.23 (95% CI= 3.08- 12.59). This could be translated into that people having the *TT* genotype are 6.23- fold more at risk of developing MS, in the multivariate logistic regression analysis (Table 1). Under the codomi-nant model of inheritance, the *CT* genotype was also associated with an increased risk for MS with an odds ratio lower than recessive model (adjusted OR= 2.9, 95% CI= 1.88- 4.49). When we combined heterozygous and homozygous variant genotypes, the adjusted OR for the *CT/TT* genotypes was 3.4 (95% CI= 2.29- 5.17). In case- case comparisons, we observed no differences in frequencies of *MTHFR C677T* genotypes in patients stratified by the clinicopathologic variables, including age, sex, and disease type (data not shown).

For the *A1298C *polymorphism, while the allele frequency distribution in MS patients was almost the same as that of controls (36.7% versus 34.7%), the genotype frequency distributions were significantly different (P = 0.001; Table 1). Analysis of the *MTHFR A1298C* frequency data obtained in this study showed that the heterozygotes (*AC*) were overrepresented in MS patients (67.8% versus 45.7%, P= 0.00) and a small trend for a higher frequency of the homozygous mutant genotype (*CC*) was observed in controls (11.8% versus 2.8%, P= 0.04). According to the logistic regression model, in the entire group of patients, *MTHFR AC* and *AC+‏CC* genotypes were strongly associated with a higher risk of MS incidence (Table 1). The adjusted OR for *AC* and *AC+CC *genotypes were 2.14 (95% CI 1.37- 3.34) and 1.77 (95%CI 1.15- 2.73), respectively. In case- case comparison, no statistically significant differences in frequencies of the *MTHFR A1298C* genotypes were found in patients stratified by age, sex, and disease type (data not shown).

**Table 1:** Distribution of *MTHFR* C677T and A1298C genotypes and alleles in MS patients and controls

**MTHFR**	**Genotypes** **& alleles**	**Patients** **N (%)**	**Controls** **N (%)**	**OR** **(95% CI)**	**P** *
C677T		180	231		
Total	CC (ref)	63 (35)	150 (65)	1	
	CT TT CT+TT C T	83 (46.1) 34 (18.9) 117 (65) 209 (58.1) 151 (41.9)	68 (29.5) 13 (5.5) 81 (35.1) 368 (79.7) 94 (20.3)	2.9 (1.88- 4.49) 6.23 (3.08-12.59) 3.44 (2.29-5.17) 1 2.83 (2.08-3.85)	**0.00** **0.00** **0.00** **0.00**
		180	186		
A1298C Total	AA (ref) AC	53 (29.4) 122 (67.8)	79 (42.5) 85 (45.7)	1 2.14 (1.37-3.34)	
	CC AC+CC A C	5 (2.8) 127 (70.6) 228 (63.3) 132 (36.7)	22 (11.8) 107 (57.5) 243 (65.3) 129 (34.7)	0.34 (0.12-0.950) 1.77 (1.15-2.73) 1 1.09 (0.81-1.48)	**0.001** **0.04** **0.010** 0.574
	CC/AA (ref)	180 22 (12.2)	186 46 (24.7)	1	
Combined genotypes (677/1298) Total	CC/AC CC/CC CT/AA **CT/AC** CT/CC **TT/AA** **TT/AC** TT/CC	39 (21.7) 2 (1.1) 17 (9.4) 63 (35) 3 (1.7) 14 (7.8) 20 (11.1) 0(0.00)	57 (30.8) 14 (7.5) 27 (14.5) 25 (13.4) 5 (2.7) 6 (3.2) 3 (1.6) 3 (1.6)	1.43 (0.75- 2.74) 0.30 (0.06- 1.43) 1.32 (0.60- 2.91) 5.27 (2.65- 10.48) 1.26 (0.28- 5.73) 4.87 (1.65-14.41) 13.94 (3.74- 51.95) ND	0.28 0.13 0.50 **0.00** 0.77 **0.004** **0.00** ND

* For OR and 95% CI calculations, controls with the wild-type *CC* and *AA MTHFR* genotypes were used as reference category. ND: not determined.

For *MTHFR *677 and 1298 combined genotypes, double heterozygotes (*677 CT/1298 AC*) had 5.3- fold (95% CI 2.65- 10.48) increased risk compared with the wild- type (*677 CC/1298 AA*) genotypes of controls (Table 1). Individuals with the *677TT/1298AC *and *677TT/1298AA *genotypes were also at higher risk of developing MS in comparison to controls. The adjusted OR for *TT/AC* and *TT/AA* combined genotypes were 13.94 (95% CI 3.74- 51.95) and 4.87 (95% CI 1.65- 14.41), respectively. Due to the small number of cases in the current study, it was not possible to perform the analyses of MS risk associated with double homozygous mutants of *677TT/1298CC *genotypes.

## Discussion

Previous studies have suggested an influence of genetic factors in the aetiology of multiple sclerosis; however, the underlying molecular mechanisms of MS remain unidentified (27, 28). It has been reported that MS patients have elevated levels of plasma and cerebrospinal fluid homocysteine, a neurotoxic metabolite (7, 10, 29-31).

*MTHFR* deficiency is the most common genetic cause of hyperhomocysteinemia (13, 16). MTHFR enzyme reduces 5, 10-methylenetetra-hydrofolate to 5-methyltetrahydrofolate, which is required for remethylation and conversion of homocysteine to methionine.

The results of previous studies with regard to the association between two polymorphisms of *MTHFR *with reduced enzyme activity (*C677T* and *A1298C*) and MS risk have been inconsistent. We examined the relationship between *MTHFR C677T* and *A1298C *polymorphisms and the risk of MS in a Southern Iranian population. According to the logistic regression model, in the entire group of patients, *MTHFR C677T *and *A1298C *polymorphisms were strongly associated with a higher risk of MS (Table 1). Compared with controls, the *MTHFRC677T* genotype showed a higher risk of MS incidence both in the recessive and codominant models (for *TT* versus *CC*: OR= 6.23, 95 % CI= 3.08-1 2.59 and *CT* versus *CC*: OR= 2.9, 95 % CI= 1.88- 4.49, respectively). We also found a higher risk associated with the *MTHFR 1298AC* genotypes when the MS patients were compared with controls and the wild- type *AA* genotype was used as a reference category (OR= 2.14, 95% CI= 1.37-3.34). However, under the recessive model of inheritance, the homozygous *CC* genotype was slightly associated with a decreased risk for MS (OR= 0.34, 95% CI= 0.12-0.950). Such result could be due to low statistical power because of the limited number of homozygous subjects (5 out of 180 cases). It has been previously reported that compared to the 677T allele, 1298 C allele has a minor effect on MTHFR activity. Neither the homozygous nor the heterozygous state of the *MTHFR *1298C genotype is associated with higher plasma homocysteine or a lower plasma folate concentration—that was observed with the homozygous 677T allele. However, double heterozygosity for both *MTHFR *mutations results in similar features as observed in homozygotes for the 677T allele (15). When we considered both *MTHFR *1298*CC *and *CT* genotypes, we found the increased risk of MS incidence associated with the *AC*/*CC* genotypes in the entire group of patients (Table 1). Our finding is consistent with most previous reports in which an increased risk of MS was observed associated with the *MTHFR A1298C* genotype (20, 32). This is in contrast to another study (21) that found no association between the *MTHFR A1298C* polymorphism and MS in Australian population. In agreement with a previous report from Iran (33), we also found increased risk of MS associated with the *MTHFR C677T *genotype. A non- significant increased MS risk associated with the *C677T *variant genotype was also reported in a group of Australian population (22). However, some studies conducted on relatively small groups of cases, observed no association between MS and *MTHFR C677T *polymorphism (20, 22, 32). Both the *C677T* and *A1298C *variant genotypes of *MTHFR* have been associated with decreased enzyme activity, with the *C677T* having a more severe effect than the *A1298C* variant. In vitro studies have shown that the 677TT and *1298CC* variant genotypes have 60% and 30% reduced enzyme activity in comparison to the wild type *MTHFR* genotypes, respectively (16, 18, 34). Inconsistency of findings across studies could be explained with the differences in study designs, sample size, genotyping methods, racial, nutritional, and other environmental factors.

To examine the joint effect of *MTHFR *677 and 1298 genotypes on MS risk, we analyzed the relationship between combined *MTHFR *SNPs at these loci and MS risk. Out of five major combined genotypes (*CC/AC*, *CT/AA*, *CT/AC*, *TT/AA*, and *TT/AC*) that constitute almost 80% of all genotypic diversity in our study group, the distribution of three genotypes was significantly different between cases and controls (Table 1). Based on our findings, 3 genotypes with the mutant alleles (*TT/AC*, *CT/AC*, and *TT/AA*) are high-risk genotypes for developing MS (13.9, 5.3, and 4.9-fold increased risk, respectively). According to a previous study, subjects with the combined *677CT/1298AC* heterozygosity had significantly higher fasting serum homocysteine levels compared to those that were *C677T* heterozygous (16). Since both *C677T* and *A1298C *mutations can influence MTHFR activity and plasma homocysteine concentrations (15, 35), it is intriguing to beleive that mutated alleles of *MTHFR *increase the risk for developing MS in a gene dose-related manner.To our knowledge, the combined effect of *MTHFR* genotypes has not been previously analyzed in MS populations and further studies are necessary to understand the dose-dependent association of *MTHFR* alleles with MS risk.
